# Phylogenomics With Hyb-Seq Unravels Korean *Hosta* Evolution

**DOI:** 10.3389/fpls.2021.645735

**Published:** 2021-07-08

**Authors:** Mi-Jeong Yoo, Byoung-Yoon Lee, Sangtae Kim, Chae Eun Lim

**Affiliations:** ^1^Department of Biology, Clarkson University, Potsdam, NY, United States; ^2^National Institute of Biological Resources, Incheon, South Korea; ^3^Department of Biotechnology, Sungshin Women’s University, Seoul, South Korea

**Keywords:** *Hosta*, Korean *Hosta*, Hyb-seq, target enrichment, phylogenomics, divergence time estimates

## Abstract

The genus *Hosta* (Agavoideae and Asparagaceae) is one of the most popular landscaping and ornamental plants native to temperate East Asia. Their popularity has led to extensive hybridization to develop various cultivars. However, their long history of hybridization, cultivation, and selection has brought about taxonomic confusion in the *Hosta* species delimitation along with their indistinguishable morphology. Here, we conducted the first broad phylogenetic analyses of *Hosta* species based on the most comprehensive genomic data set to date. To do so, we captured 246 nuclear gene sequences and plastomes from 55 accessions of Korean *Hosta* species using the Hyb-Seq method. As a result, this study provides the following novel and significant findings: (1) phylogenetic analyses of the captured sequences retrieved six species of *Hosta* in South Korea compared to five to eleven species based on the previous studies, (2) their phylogenetic relationships suggested that the large genome size was ancestral and the diversification of Korean *Hosta* species was accompanied by decreases in genome sizes, (3) comparison between nuclear genes and plastome revealed several introgressive hybridization events between *Hosta* species, and (4) divergence times estimated here showed that *Hosta* diverged 35.59 million years ago, while Korean *Hosta* species rapidly diversified during the late Miocene. Last, we explored whether these genomic data could be used to infer the origin of cultivars. In summary, this study provides the most comprehensive genomic resources to be used in phylogenetic, population, and conservation studies of *Hosta*, as well as for unraveling the origin of many cultivars.

## Introduction

The genus *Hosta* Tratt. is an economically important group in terms of ethnobotany and horticulture. Some *Hosta* species have been cultivated for medicinal purposes since they are known to produce phytochemicals having antioxidant and anti-inflammatory activities (Mimaki et al., 1997; Yang et al., 2017; Chu et al., 2019). Many *Hosta* species are also popular as landscaping and ornamental plants because of their astonishing foliage and shade tolerance. Thus, they have been extensively hybridized to develop various cultivars, resulting in about 6,000 cultivars in the world (The_American_Hosta_Society, 2020). However, their long history of hybridization, cultivation, and selection along with their indistinguishable morphologies has brought about taxonomic confusion in *Hosta* species delimitation. Therefore, as few as 23 species (Zonneveld and Van Iren, 2001) or as many as 40 species (Fujita, 1976; Schmid, 1991; Chen and Boufford, 2000; Tamura and Fujita, 2013) have been reported from temperate East Asia, i.e., China, Japan, South Korea, and Russia.

Taxonomically, *Hosta* belongs to the Agavoideae bimodal karyotype clade (ABK clade) of Asparagaceae and diverged at the crown node of the ABK clade during the Oligocene (∼20–28 million years ago, Ma) (McKain et al., 2012, 2016). However, since only one *Hosta* species was included in those studies, further analyses with more *Hosta* species are required to estimate more accurate divergence times for *Hosta*.

The most recent taxonomic revision of *Hosta* was done based on pollen viability and nuclear DNA content, by supplementing the previous work, and it proposed three subgenera: *Hosta*, *Bryocles*, and *Giboshi* (Zonneveld and Van Iren, 2001). Subgenus *Hosta* is composed of one species, *H. plantaginea* (Lam.) Asch., while *Bryocles* and *Giboshi* contain nine species from China and South Korea and 13 species from Japan and the Russian Far East, respectively. They further divided subgenera into several sections according to their geographical distribution and nuclear DNA contents. However, their treatment did not correspond to sections previously proposed (Maekawa, 1940; Schmid, 1991), which several lines of evidence supported, such as plastid DNA (Chung, 1991), isozymes (Chung et al., 1991a), pollen morphology (Chung and Jones, 1989), and morphological characters (Chung et al., 1991b). For example, Zonneveld and Van Iren (2001) placed *H. clausa* Nakai and *H. ventricosa* (Salisb.) Stearn in different sections, but these two species were considered to have descended from a common ancestor based on pollen morphology (Chung and Jones, 1989) and karyotypes (Kaneko, 1968). Thus, further investigation of the taxonomic treatment of *Hosta* is required.

In Korea, five to eleven species have been described based on morphological characteristics, such as rhizome, petiole color, inflorescence, and floral shape (Chung and Chung, 1988; Chung and Kim, 1991; Lee, 1996; Chung, 2007; Jo and Kim, 2017), and all of them belong to subgenus *Bryocles* (Zonneveld and Van Iren, 2001). However, the lack of distinct traits with taxonomic value makes it hard to reach a consensus regarding the entity of *Hosta* species. Moreover, continuous variation in morphological traits and frequent hybridization have been problematic with regards to species delimitation. For example, *H. venusta* F. Maek. has been recognized as a species endemic to Mt. Halla (Jeju Island, South Korea), with its entity confirmed by several studies (Chung, 1990; Chung et al., 1991a,b). Notably, it was inferred that *H. venusta* might have recently derived from *H. minor* (Baker) Nakai based on morphology and distribution patterns (Chung, 1990; Chung et al., 1991a,b). In addition, Sauve et al. (2005) differentiated these two species relying on ten random amplified polymorphic DNA (RAPD) markers. However, Jo and Kim (2017) treated *H. venusta* as a variety of *H. minor* based on its morphological characteristics, overlapping with those of *H. minor*. Thus, investigating on whether these two species are distinct requires more genomic data.

To date, Chung and colleagues have performed the most comprehensive work on Korean *Hosta* species (Chung, 1990; Chung et al., 1991b). Their studies on *Hosta* from 45 populations recognized six species based on morphometric multivariate analysis and distribution patterns. Based on gross morphology, *H. yingeri* S. B. Jones and *H. capitata* (Koidz.) Nakai were distinguished from all other taxa, while *H. clausa* was differentiated from the other three taxa by the shape of its rhizome. The remaining three species, *H. minor*, *H. venusta*, and *H. jonesii* M.G. Chung, were separated by the length of their floral lobes and leaf blades. Later, based on the variation of six isozymes, Chung et al. (1991a) confirmed four groups of species: (1) *H. minor*, *H. venusta*, and *H. jonesii*, (2) *H. yingeri*, (3) *H. capitata*, and (4) *H. clausa*. Their species delimitation was mainly based on the similarity of morphological traits and a small set of isozyme data; however, their evolutionary relationships were not inferred.

Recently, the phylogenetic relationships of the six Korean *Hosta* species were reconstructed based on complete plastomes (Lee et al., 2019). This study distinguished six species, relying on four genic and intergenic regions, but nucleotide diversity among these species was very low (an average of 0.07% from pairwise comparison). The phylogenetic analysis showed that three species, *H. minor*, *H. venusta*, and *H. jonesii* are closely related. Additionally, they found that *H. yingeri* formed a clade with *H. jonesii*, which conflicts with previous studies (Chung, 1990; Chung et al., 1991b). However, as their work included one plastome per species, population level sampling and sequence data from the nuclear genome are needed to evaluate phylogenetic relationships of Korean *Hosta* species and to investigate whether hybridization has occurred or not.

Recent progress in high-throughput sequencing (HTS) subsampling methods has enabled us to evaluate phylogenetic relationships of species much more efficiently, by increasing the number of nuclear genes that can easily and inexpensively be sequenced (McKain et al., 2018). Notably, Hyb-Seq, a modified target sequencing method, is an effective way to capture DNA sequences from many single-to-low-copy nuclear genes, as well as a way to obtain regions in high-copy number, i.e., organellar genomes (Weitemier et al., 2014). Since this organellar DNA can provide important information for reticulate evolution and introgression, having both nuclear and organellar DNA makes Hyb-Seq a very powerful approach.

Here, we used Hyb-Seq to capture 246 single-to-low-copy nuclear gene sequences and complete plastomes. We focused on Korean *Hosta* species to test whether these captured genomic resources possessed sufficient phylogenetic signal that could help unravel *Hosta* evolution. Therefore, we included 55 accessions representing six Korean *Hosta* species and two Chinese ones and aimed to answer the following questions: (1) What are the phylogenetic relationships for these *Hosta* species inferred from multiple accessions based on both nuclear and plastid sequences? (2) Are nuclear and plastid phylogenetic relationships congruent with each other? (3) When did Korean *Hosta* species diverge? Lastly, (4) Are these genomic data appropriate to infer the origin of extant cultivars? Considering many putative *Hosta* species are cultivars maintained through time via vegetative propagation, this study provides a cornerstone for the development of potential molecular markers to unravel the origin of said cultivars, as well as their entity, which can be of use in breeding programs.

## Materials and Methods

### RNA Extraction, Library Preparation, and Sequencing

To select single-to-low-copy nuclear genes for target capture probe design, we first profiled the leaf transcriptome of *H. venusta.* Total RNAs from leaf tissue were isolated, with the RNeasy Plant Mini Kit (Qiagen, Hilden, Germany), and treated with Ribo-Zero rRNA Removal kit (Plant leaf) (Illumina, San Diego, CA, United States). RNA was quantified with a Victor2 spectrophotometer (PerkinElmer, Shelton, CT, United States) using the Quant-iT^TM^ RiboGreen^TM^ RNA Assay Kit (Invitrogen, Carlsbad, CA, United States). A total of 4 ng RNA was used for library preparation with the TruSeq Stranded Total RNA Sample Preparation kit (Illumina, San Diego CA, United States) and an alternate fragmentation protocol (fragmentation at 94°C for 2 min) was followed to obtain insert sizes of 280 bp, as described in the manufacturer’s protocol. The obtained libraries were quantified using the Agilent DNA 1000 Kit (Agilent, Santa Clara, CA, United States) and the Ssofast^TM^ EvaGreen supermix (Bio-Rad Laboratories Inc., Hercules, CA, United States). A final library concentration of 3 nM was sequenced on an Illumina NextSeq 500 sequencer (Illumina, San Diego, CA, United States), with 150 paired-end sequencing (2 × 150 bp), at Macrogen (Seoul, South Korea). Reads generated from the transcriptome of *H. venusta* were assembled *de novo* using Trinity v2.2.0 (Grabherr et al., 2011) with default options.

### Probe Design

To determine target genes and to design probes, we first assembled *H. venusta* transcripts using 29,222,496 filtered reads; fastq files with raw reads (R1 and R2) were deposited to the GenBank Sequence Read Archive (PRJNA673211). The assembled *H. venusta* transcriptome was compared to the proteomes of *Arabidopsis thaliana*, *Populus trichocarpa*, *Oryza sativa*, and *Vitis vinifera*, as references, using the MarkerMiner pipeline (ver. 1.0) (Chamala et al., 2015). As a result, 283 orthologous genes (494,539 bp of total exon length) were selected for probe design based on GC content (40∼60%), number of hits (≤3), and E-value (≤1E–28) from BLAST searches against the *Asparagus* genome (Harkess et al., 2017), which is the closest relative to *Hosta* among fully sequenced plant genomes. The comparison of our target genes with a commercially available universal gene capture enrichment panel, Angiosperm353, resulted in only 30 overlapping genes at both nucleotide and protein levels. The final 4,247 probes (each 120 bp long; an average of 15 probes per gene) were synthesized at Agilent Technologies (Santa Clara, CA, United States) with 3× tiling across target exons. These probes targeted 676 exons (171,365 bp) covering 283 genes (436 kb of coding regions) total.

### DNA Extraction, Library Preparation, In-Solution Hybridization, and Sequencing

**FIGURE 1:**
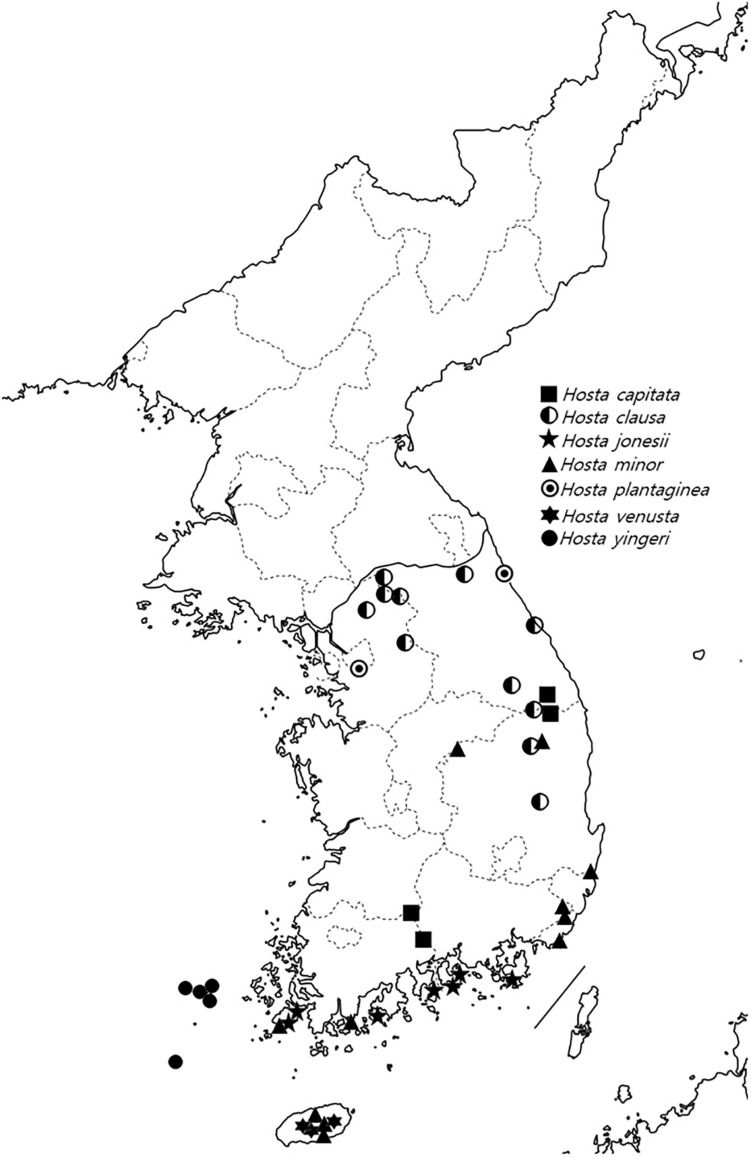
Distribution of Korean *Hosta* species used in this study.

We sampled 64 accessions belonging to seven species of *Hosta*, including nine cultivars (Figure 1 and Supplementary Table 1). Voucher specimens for these samples were deposited at the herbarium of the National Institute of Biological Resources (NIBR), Incheon, South Korea. DNAs were extracted from either silica-gel-dried or herbarium material using a DNeasy Plant Mini Kit (Qiagen, Hilden, Germany) and were quantified using a Quant-iT^TM^ PicoGreen^TM^ dsDNA Assay Kit (Invitrogen, Carlsbad, CA, United States) with a Synergy^TM^ LX Multi-Mode Microplate Reader (BioTek, Winooski, VT, United States). Libraries were prepared from a total of 50 ng of DNA using the TruSeq^®^ Nano DNA Library Prep kit (Illumina, San Diego CA, United States) with 2 × 8 bp dual indexes to allow for multiplexed sequencing. After quantification, equimolar samples were pooled (16 libraires per reaction), hybridized with biotin-labeled baits to retrieve our targets using the probe kit described above. Captured DNA fragments were enriched in a 14–16 PCR cycle to generate the final libraries. A final concentration of 3 nM library was pooled and sequenced on an Illumina HiSeq 3000 (2 × 150 bp) (Illumina, San Diego, CA, United States) at RAPiD Genomics (Gainesville, FL, United States).

### Contig Assembly and Multi-Sequence Alignment

Filtering of low-quality reads and adapter trimming were conducted by Skewer (v0.2.2) (Jiang et al., 2014) with options (mq = “30”, st = “3”, er = “0.1”, ml = “25”, a1 = “AGATCGGAAGAGCACACGTCTGAACTCCAGTCA”, a2 = “AGATCGGAAGAGCGTCGTGTAGGGAAAGAGTGT”). We employed HybPiper pipeline v.1.2 (Johnson et al., 2016) to process the trimmed data, using the BWA aligner (Li and Durbin, 2009) and the SPAdes v.3.10.1 assembler (Bankevich et al., 2012). Assembled contigs containing both on-target exons and off-target partial introns were combined into supercontigs. Potential paralogs flagged by the HybPiper paralog_investigator.py script were excluded from further analyses. Each supercontig was aligned using MAFFT v.7.402 (Katoh et al., 2002). Because the alignment included sample-specific indels, we removed them via Phyutility v.2.2.6 (Smith and Dunn, 2008) using -clean 0.7 option. Each cleaned supercontig file was concatenated, when necessary, according to sample ID. A total of 246 genes was used in downstream analyses.

### Genome Skimming and Plastome Assembly

For plastome assembly, 64 genomic DNA libraries were sequenced on an Illumina HiSeq 3000 (2 X 150 bp), and the reads were trimmed as described above. Reconstruction of the plastome was performed using MITObim v.1.9.1 (Hahn et al., 2013). In short, the reads derived from a plastome were retrieved by mapping the trimmed reads onto the plastome sequence of *H. minor* (MK732316). Then, an initial reference for the species in question was built from the mapping result. The reads with overlap to this initial reference were fished, and these reads subsets were mapped to said initial reference. Thus, the reference extended iteratively by repeatedly fishing and mapping until all gaps were closed and the number of reads remained stationary. The assembled sequences were reevaluated by mapping the reads to previously published *Hosta* plastome sequences (Lee et al., 2019) using Geneious v.2019.2.3^1^.

### Sequence Diversity and Phylogenetic Inference

To estimate how much genetic diversity exists within and between *Hosta* species, we calculated nucleotide diversity (π) and genetic differentiation (*F*_*st*_) among species using DnaSP v.6.12.03 (Rozas et al., 2017). Nuclear and plastid phylogenies of *Hosta* were inferred from the cleaned concatenated nuclear and plastome data matrices, respectively. Molecular evolution models were evaluated using ModelFinder (Kalyaanamoorthy et al., 2017), and maximum likelihood (ML) trees were inferred in IQ-TREE v.1.6.11 (Nguyen et al., 2015) with the standard nonparametric bootstrap method (-b 1000). Four plastome sequences of *Yucca queretaroensis* (NC_032713.1), *Y. brevifolia* (NC_032711.1), *Agave attenuata* (NC_032696.1), and *Hesperoyucca whipplei* (NC_032705.1) were used as outgroups in the plastome ML analysis, while the nuclear concatenated ML tree was rooted using two *H. plantaginea* species since they were inferred as sister to the remaining taxa in the plastome ML tree. We also inferred a species tree using the multispecies coalescent model, in which individual nuclear gene trees were constructed using RAxML v.8.2.12 under the GTRCAT substitution model and 200 bootstrap replicates by slow ML optimization (Stamatakis, 2014). Then ASTRAL-III v.5.6.2 (Zhang et al., 2018) was run to obtain a species tree, along with quartet support values that were visualized as pie charts relying on M. G. Johnson’s script^2^. We further evaluated gene conflict by measuring gene concordance factors (gCF) and site concordance factors (sCF) using IQ-TREE v.2.1.0 (Nguyen et al., 2015; Minh et al., 2020). It is thought that the plastome evolves as a single locus at a relatively constant rate, but we investigated whether this assumption holds for *Hosta* species by partitioning the plastome dataset into 130 genes and 117 intergenic areas to calculate gCF and sCF. Finally, the concatenated ML tree obtained from nuclear data was employed to reconstruct genome size ancestral states for *Hosta* species using the R package phytools v.0.7-70 under Brownian motion (Revell, 2012). The genome size data were obtained from Zonneveld and Van Iren (2001). Tree files, datasets and code used in R v.4.0.2 (R Core_Team, 2000) are available through the figshare data repository^3^.

### Divergence Time Estimation

To estimate divergence times for Korean *Hosta* species, we only considered the plastome. Although nucleotide diversity was higher among nuclear genes, we were concerned rate heterogeneity among-sites and among-lineages, and recombination within and between loci, could severely impact divergence time estimates. Additionally, the 246 nuclear genes obtained here exhibited high among-gene incongruence (normalized quartet score = 0.61). Ideally, a wide range of outgroups with well-known fossils would be needed to calibrate our data matrices; however, those outgroups are missing from our nuclear dataset. Therefore, we employed the plastome which, in theory, acts as a single locus with no recombination and relatively homogenous evolutionary rate. A total of 79 protein-coding plastome genes (*accD, atpA, atpB, atpE, atpF, atpH, atpI, ccsA, cemA, clpP, infA, matK, ndhA, ndhB, ndhC, ndhD, ndhE, ndhF, ndhG, ndhH, ndhI, ndhJ, ndhK, pbf1, petA, petB, petD, petG, petL, petN, psaA, psaB, psaC, psaI, psaJ, psbA, psbB, psbC, psbD, psbE, psbF, psbH, psbI, psbJ, psbK, psbL, psbM, psbT, psbZ, rbcL, rpl2, rpl14,rpl16,rpl20, rpl22, rpl23, rpl32, rpl33, rpl36, rpoA, rpoB, rpoC1, rpoC2, rps11, rps12, rps14, rps15, rps16, rps18, rps19, rps2, rps3, rps4, rps7, rps8, ycf1, ycf2, ycf3*, and *ycf4*), commonly conserved in all taxa surveyed, were employed from 40 taxa of Asparagales, including 16 *Hosta* accessions (Supplementary Table 2). The aligned sequences contained 68,766 bp, and a ML phylogeny was inferred as described above. Since the monophyly of each subfamily and the genus *Hosta* was confirmed (Supplementary Figure 1), we constrained these clades for the BEAST 2 run. Divergence times were estimated using BEAST v.2.6.2 (Bouckaert et al., 2019) after generating BEAST input XML files using BEAUti v2.6.2. We followed the model parameters and the method used in McKain et al. (2016). In short, a GTRGAMMA evolutionary model was used with 10 gamma rate categories. A log-normal relaxed clock (Drummond et al., 2006) was the clock model, using the ML tree estimated above as the starting tree, and with speciation modeled by the Yule process. Age calibrations were implemented as follows: a log-normal distribution with a mean of 1.0, a standard deviation of 1.0, and an offset of 11.28 for the crown group of *Yucca* (Tidwell and Parker, 1990) and a log-normal distribution with a mean of 1.0, a standard deviation of 1.0, and an offset of 61.33 for *Anemarrhena* (McKain et al., 2016). We ran five separate runs for a total of 50 million generations, sampling every 1000. Convergence was assessed with Tracer v.1.7.1, and runs were continued to reach effective sample size (ESS) values of at least 200. Runs were combined using LogCombiner v.2.6.2 with a 10% burnin, and a maximum clade credibility (MCC) tree with mean node heights was created using a sample of 22,424 trees in TreeAnnotator v.2.6.2. The chronogram was drawn using FigTree v.1.4.3^4^.

## Results

### Target Nuclear Dataset

*De novo* assembly of *H. venusta* transcripts resulted in 68,043 clustered unigenes, with a total of 1,013 transcripts as putative single-copy orthologs to four species. Among these, 283 transcripts, identified as putative orthologs, for at least three reference species, were selected as target genes. After target enrichment and sequencing, nine out of 64 accessions were excluded due to their low coverage (<5%). As for 55 accessions, an average of 4,982,380 reads were obtained per sample, and 74.5% of them were mapped to target genes (Supplementary Table 1). Average reads mapping on target equaled 680.67 (minimum = 325.19, maximum = 2688.39), and the general error rate (sequence divergence compared to the reference) was 2.84% on average (Supplementary Table 1). In agreement with using transcripts from *H. venusta* as the reference transcriptome, *H. venusta* (Hosven_15311) showed the least sequence divergence (2.40%), while *H. yingeri* (Hosyin_0616_1) exhibited the highest sequence divergence (3.32%) (Supplementary Table 1). These sequence divergences reflect their respective phylogenetic distances. For example, *H. minor* and *H. venusta* exhibited similar sequence divergences of 2.65% on average, while *H. yingeri* showed the highest degree of sequence divergence among seven species, supporting its isolation from the other species. We analyzed potential paralogs to evaluate their effect on phylogenetic inference, and we observed that relationships inferred were not congruent. Since high discordance was observed among ortholog genes, probably due to a rapid radiation in *Hosta* species (see below), we excluded paralogs from further analyses. After the data clean-up and the exclusion of paralogs, a total of 246 nuclear genes (489,796 bp) were included in the final alignment. The length of assembled sequences ranges from 359,467 bp in *H. minor* (Hosmin_15305_3) to 480,309 bp in *H. clausa* (Hoscla13), with an average of 3.67% gaps in the final alignment (Supplementary Table 1). When recovered sequences were parsed into exons and flanking regions, an average of 223 kb corresponded to exon regions, equal to 130% of target exons (171 kb, meaning their length was greater than expected) and 51% of target genes (436 kb of coding regions). Also, our designed probe set captured an additional average of 211 kb of flanking non-coding regions, including introns and 5′ or 3′-untranslated regions (UTRs). The final alignment was deposited to the figshare data repository (see text footnote 3).

### Plastome Assembly

The genome skimming resulted in an average of 2,521,082 filtered reads per sample. An average of 2.25% filtered reads was mapped to their own assembled references, ranging from 0.20% in *H. yingeri* (Hosyin_16285) to 7.19% in *H. clausa* (Hoscla_16) (Supplementary Table 1). The size of assembled plastomes ranges from 154,648 bp in *H. yingeri* (Hosyin_16285; 98.7% of the complete plastome) to 156,756 bp in *H. yingeri* (Hoysin_s_n; 100% of the complete plastome). Previously reported sequence variation in the intergenic region between *trnK-UUU* and *trnQ-UUG* (Lee et al., 2019) was observed; that is, a 278 bp-deletion was found in all *H. capitata* species investigated here, except for a single cultivar (Hoscap72). Compared to four outgroup taxa, plastome sequences of *Hosta* species (156,455 bp on average) were shorter (157,776 bp on average), due to the loss of exon 2 of the *rps16* gene, as previously known from *H. ventricosa* (Steele et al., 2012; McKain et al., 2016). The final alignment (159,590 bp) was also stored in the figshare data repository (see text footnote 3).

### Sequence Divergence and Genetic Differentiation

We examined sequence divergence by calculating nucleotide diversity (π) within and among species groups. In general, the nucleotide diversities of nuclear transcripts were much higher than those of plastome sequences (Table 1). The nucleotide diversity for each species ranges from 0.17% (*H. clausa*) to 0.91% (*H. venusta*) across the 246 nuclear target genes, while it ranges from 0% (*H. plantaginea*) to 0.02% (*H. clausa*) for the plastome sequences (Table 1). Among species, the nucleotide diversity between *H. plantaginea* and *H. ventricosa* showed the highest value in both nuclear DNA (2.7%) and plastid data (0.23%) (Table 1). Meanwhile, the nucleotide diversity between *H. minor* and *H. venusta* was 1.2% and 0.004% for the nuclear and plastid data, respectively, but *H. venusta* accessions were embedded within the *H. minor* clade in both ML trees (see below).

**TABLE 1:** Nucleotide diversity (π) among and between *Hosta* species.

(A) Nucleotide diversity based on 246 nuclear targets (489,796 bp).								

	*H. minor*	*H. venusta*	*H. jonesii*	*H. clausa*	*H. capitata*	*H. yingeri*	*H. ventricosa*	*H. plantaginea*
*H. minor* (11)	0.00831							
*H. venusta* (5)	0.01210	0.00913						
*H. jonesii* (6)	0.01330	0.01708	0.00856					
*H. clausa* (11)	0.01345	0.01526	0.01438	0.00625				
*H. capitata* (4)	0.01417	0.01863	0.01691	0.01381	0.00387			
*H. yingeri* (5)	0.01612	0.01867	0.01782	0.01549	0.01740	0.00685		
*H. ventricosa* (2)	0.01442	0.02067	0.01841	0.01336	0.01442	0.01855	0.00627	
*H. plantaginea* (2)	0.01614	0.02372	0.02047	0.01472	0.02045	0.01990	0.02700	0.00174

**(B) Nucleotide diversity based on plastome sequences (159,590 bp).**								

	***H. minor***	***H. venusta***	***H. jonesii***	***H. clausa***	***H. capitata***	***H. yingeri***	***H. ventricosa***	***H. plantaginea***

*H. minor* (12)	0.00004	5.542	47.000	74.493	68.000	39.176	42.055	122.560
*H. venusta* (6)	0.00004	0.00001	55.818	71.860	75.758	43.273	61.750	214.321
*H. jonesii* (5)	0.00031	0.00036	0.00016	74.431	96.909	39.273	104.190	265.524
*H. clausa* (13)	0.00049	0.00046	0.00048	0.00018	77.053	71.860	62.171	141.676
*H. capitata* (6)	0.00044	0.00049	0.00062	0.0005	0.00005	83.576	50.286	222.929
*H. yingeri* (6)	0.00026	0.00028	0.00026	0.00046	0.00055	0.00001	74.214	210.607
*H. ventricosa* (2)	0.00027	0.00040	0.00067	0.00040	0.00032	0.00048	0.00031	362.833
*H. plantaginea* (2)	0.00080	0.00137	0.00170	0.00091	0.00143	0.00137	0.00233	0.00001

*The numbers in parentheses next to the species names indicate the number of taxa sampled per species.*

We also investigated genetic differentiation among species. In general, estimated *F*_*st*_ values were higher for plastome sequences than for nuclear transcripts (Table 2). Most species had high *F*_*st*_ values (> 0.25), indicating significant genetic differentiation between species. However, *H. minor* and *H. venusta* accessions showed little genetic differentiation (plastome *F*_*st*_ = 0.2123, nuclear *F*_*st*_ = 0.0686) (Table 2).

**TABLE 2:** Genetic differentiation (*F*_*st*_) between *Hosta* species based on 246 nuclear targets and plastome sequences.

	*H. minor*	*H. venusta*	*H. jonesii*	*H. clausa*	*H. capitata*	*H. yingeri*	*H. ventricosa*	*H. plantaginea*
*H. minor*	–	0.0686	0.2966	0.4864	0.5625	0.5932	0.4647	0.7426
*H. venusta*	0.2123	–	0.3106	0.4823	0.5712	0.5877	0.4749	0.7310
*H. jonesii*	0.8372	0.8564	–	0.4715	0.5537	0.5850	0.4944	0.7372
*H. clausa*	0.8642	0.8794	0.8036	–	0.6605	0.6579	0.5823	0.7885
*H. capitata*	0.9455	0.9612	0.8989	0.8608	–	0.7123	0.6701	0.8575
*H. yingeri*	0.9476	0.9773	0.7744	0.8701	0.9660	–	0.6737	0.8081
*H. ventricosa*	0.8010	0.8135	0.8033	0.7482	0.7060	0.8493	–	0.8095
*H. plantaginea*	0.9913	0.9966	0.9718	0.9658	0.9892	0.9973	0.9475	–

*The numbers diagonally downward and upward indicate F_st_ obtained from the plastome sequences and nuclear targets, respectively.*

### Phylogenetic Inference

We investigated phylogenetic relationships among seven Korean *Hosta* species using 246 single-copy nuclear orthologs and the whole plastome. The concatenated nuclear alignment included 54,754 parsimony informative sites (PIS, out of 489,796 bp, i.e., 11.2%), while the plastome sequence alignment contained 1,851 PIS (out of 159,590 bp, i.e., 1.2%).

**FIGURE 2:**
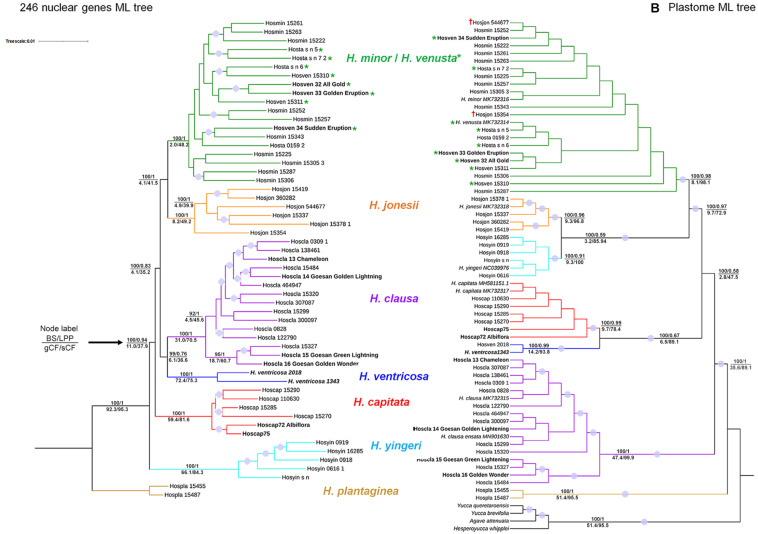
Comparison of maximum likelihood trees based on **(A)** nuclear DNA data matrix (246 loci, 489,796 bp) with a model of GTR + F + R3 and **(B)** plastome sequence matrix (159,590 bp) with a model of K3Pu + F + R2. The plastome tree has very short branch lengths for most clades (Supplementary Figure 2), so a cladogram was presented here. In both trees, the upper numbers on the node represent bootstrap support (BS) from 1,000 replicates for the concatenated dataset with IQ-TREE and local posterior probability (LPP) from the ASTRAL species tree, while the lower numbers indicate gene concordance factor (gCF) and site concordance factor (sCF) values. The values were not shown for branches in conflict between IQ-TREE and ASTRAL-III trees. In both trees, purple dots indicate ≥ 95 bootstrap support (BS). Bold taxa show cultivated species, while green asterisks indicate *H. venusta*. † shows *H. jonesii* accession which has the *H. minor*-like plastome.

Maximum likelihood (ML) and ASTRAL trees from both nuclear and plastome sequences showed congruence in terms of retrieving seven putative species: (1) *H. minor*/*H. venusta*, (2) *H. jonesii*, (3) *H. clausa*, (4) *H. capitata*, (5) *H. ventricosa*, (6) *H. yingeri*, and (7) *H. plantaginea*; all of them received maximum bootstrap support (BS) (Figure 2 and Supplementary Figures 2, 3). Although BS is 100% and local posterior probability (LPP) is 1 for these clades, underlying gene tree conflict seems to be extensive, for example, for the *H. jonesii* clade gCF is 8.2%, with 49.2% sCF, in the nuclear tree (Figure 2A). These values show that only 8.2% of 246 gene trees and 49.2% of phyloinformative alignment sites support this clade. In the ASTRAL tree, only 21 of 246 gene trees showed concordance for the same clade (Supplementary Figure 3), while gCF obtained from the plastome sequences was 9.3%, while sCF was 96.8% (Figure 2B).

In the plastome ML tree, *H. plantaginea* was inferred to be sister to the remaining species, as supported by 51% gCF and 95.5% sCF, along with 100% BS and 1 LPP (Figure 2B and Supplementary Figure 2). Branches subtending both *H. plantaginea* and the clade composed of the remaining *Hosta* species are notably long (Supplementary Figure 2). The sister relationship between *H. plantaginea* and the others was also observed in the nuclear ML tree and was overall highly supported (Figure 2).

Two species, *H. minor* and *H. venusta*, were not reciprocally monophyletic. In the nuclear ML tree, seven of eight accessions of *H. venusta* formed two clades nested within a *H. minor* clade, while the remaining accession (Hosven_34) was sister to a different *H. minor* accession (Hosmin_15343). In the plastome ML tree, *H. venusta* accessions were interspersed along a *H. minor* clade (Figure 2). This clade was defined by a high degree of gene tree discordance, for example, only 6 out of 246 gene trees support their grouping (Supplementary Figure 3).

These two ML trees showed other disparities. For example, *H. yingeri* was sister to the remaining species, other than *H. plantaginea*, in the nuclear ML tree, while this same species was sister to a *H. jonesii* clade in the plastome ML tree (Figure 2). Also, two *H. jonesii* accessions (Hosjon_544677, Hosjon_15354) were nested in the *H. minor*/*H. venusta* clade in the plastome ML tree (Figure 2). These accessions formed a clade with *H. minor*/*H. venusta* in some trees obtained from the partitioned analysis of nuclear data (Supplementary Figure 4). In addition, three species clades, *H. clausa*, *H. capitata*, and *H. ventricosa*, exhibited topological differences between the nuclear and plastome trees. In the nuclear tree, *H. capitata* was sister to all other species, except for *H. yingeri* and *H. plantaginea*, while *H. clausa* was sister to *H. ventricosa*, and both were sister to the clade including *H. minor*, *H. venusta*, and *H. jonesii*. In the plastome tree, however, a clade of *H. clausa* species was sister to all other species except for *H. plantaginea*, while *H. capitata* was sister to *H. ventricosa* (Figure 2). These overall disparities can be explained by incongruence, as shown by the low values of gCF (Figure 2) and quartet support values for the clades (Supplementary Figure 3), as well as by the low normalized quartet score (NQS) values for the nuclear genes (0.61) and the plastome sequences (0.43). However, the NQS value for the plastome sequences was much lower than the one estimated by Gonçalves et al. (2019) for 89 rosid species (0.8), probably due to the lack of phylogenetic signal resulting from the low genetic diversity (Table 1).

**FIGURE 3:**
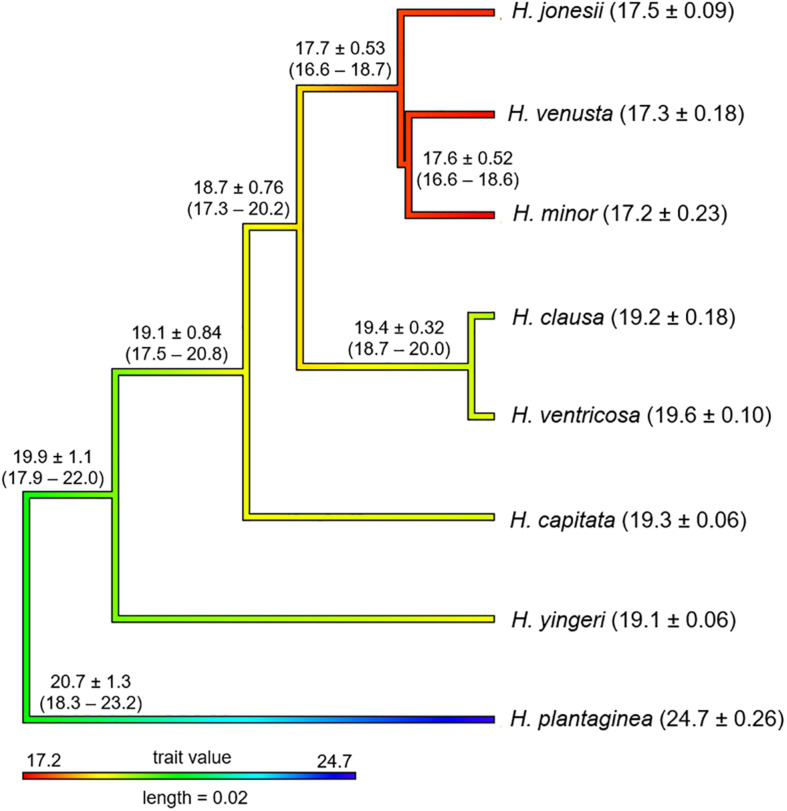
Ancestral character reconstruction of genome size evolution along the branches and nodes of the tree obtained from 246 nuclear target genes. The numbers in parentheses next to the species names show nuclear DNA content (2C) (average ± standard deviation) from Zonneveld and Van Iren (2001). The 2C value was reduced to half in *H. ventricosa* (2*n* = 4*x* = 120). The numbers near the branches and nodes represent the ancestral character estimates ± standard deviation with lower and upper 95% of confidence intervals.

Using the tree inferred from nuclear DNA data, we reconstructed the genome size ancestral state in *Hosta*. The result points to large genome size (19.9 ± 1.1 pg) as the ancestral state for the lineage leading to Korean *Hosta* species that have smaller genome sizes, while the *H. plantaginea* clade seems to have experienced an increase in its genome size sometime during its evolutionary history (Figure 3).

To evaluate the application of the genomic resources generated here for inferring the origin of cultivars, we included several cultivar accessions. Since our sampling is limited to *Hosta* species in South Korea, we utilized nine cultivars for which their parentage is known. All these cultivars were clustered with their parental species, for example, cultivar “Chameleon” (Hoscla_13) was nested in the *H. clausa* clade, while cultivar “Albiflora” (Hoscap72) was nested in the *H. capitata* clade (Figure 2), their known respective parentals.

### Divergence Time Estimates

**FIGURE 4:**
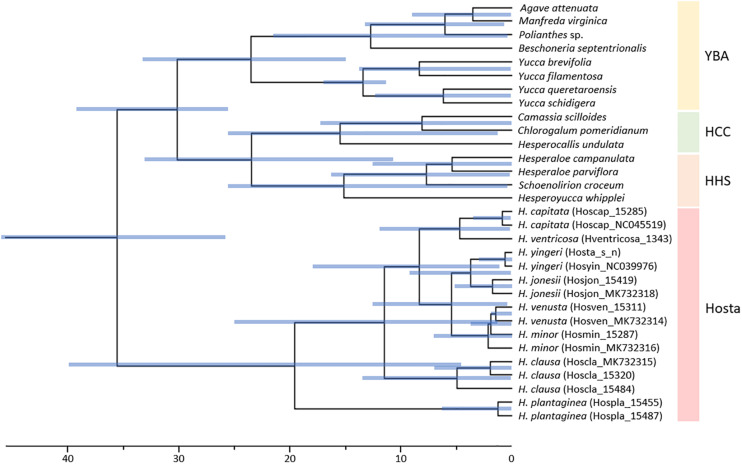
Divergence time estimate for Korean *Hosta* species inferred from 79 protein-coding plastome genes. Blue bars represent 95% highest posterior density (HPD) intervals. YBA, *Yucca-Beschorneria-Agave*; HHS, *Hesperoyucca-Hesperaloe-Schoenolirion*; HCC, *Hesperocallis-Camassia-Chlorogalum.*

To estimate divergence times for Korean *Hosta* species, we used 79 plastome protein-coding genes from 40 Asparagales taxa relying on two calibration points, and the purported ages of *Yucca* and *Anemarrhena*. Estimated divergence times for lineage splits were different from those of McKain et al. (2016). We inferred the stem age of *Hosta* at 35.59 Ma (late Eocene) (95% highest posterior density (HPD) interval: 25.94–46.23 Ma) (Figure 4 and Table 3), while McKain et al. (2016) estimated this same stem age at 27.92 Ma (middle Oligocene) (95% HPD interval: 37.51–20.70 Ma). Here, we first report a divergence time estimate for the *Hosta* crown group and the Korean *Hosta* species. The *Hosta* crown group diverged 19.60 Ma (early Miocene), while the Korean species diverged during the late Miocene (11.51 Ma for *H. clausa*, 3.74 Ma for *H. jonesii*) (Figure 4 and Table 3).

**TABLE 3:** Divergence time estimates for Korean *Hosta* and related lineages.

Split	Age (lower 95% HPD – upper 95% HPD) (Mya)	Age (lower 95% HPD – upper 95% HPD) (Mya) McKain et al. (2016)
*Anemarrhena*	65.74 (61.33–78.99)	61.33 (47.60–75.81)
*Behnia-Hosta*	46.00 (29.84–59.59)	41.27 (26.37–55.83)
YBA + HHS - HCC	30.17 (25.75–39.43)	25.75 (18.42–34.50)
*Yucca*	23.45 (15.11–33.36)	20.10 (14.44–27.09)
*Yucca* crown	15.13 (11.40–17.06)	12.45 (11.35–14.57)
HHS-HCC	23.51 (10.71–33.28)	21.92 (14.80–29.5)
*Hosta*	35.59 (25.94–46.23)	27.92 (20.70–37.51)
*Hosta* crown	19.60 (4.61–40.07)	–
*H. clausa*	11.51 (1.41–25.25)	–
*H. capitata + H. ventricosa*	8.34 (1.20–18.10)	–
*H. minor/H. venusta*	5.43 (0.40–12.60)	–
*H. jonesii*	3.74 (0.17–9.23)	–

*YBA, Yucca-Beschorneria-Agave; HHS, Hesperoyucca-Hesperaloe-Schoenolirion; HCC, Hesperocallis-Camassia-Chlorogalum.*

## Discussion

### Species Entity and Phylogenomic Relationships of Korean *Hosta* Species

Previously, many researchers have proposed evolutionary relationships for Korean *Hosta* species, but those were based on limited information. Recently, Lee et al. (2019) employed whole plastome sequences, but only included an individual per species in their analysis. Therefore, this study provides the first instance of phylogenetic inference based on comprehensive data for most genomic compartments: 246 single-to-low-copy nuclear genes and whole plastome sequences from 55 accessions of Korean *Hosta* species.

In both nuclear and plastome ML trees, *H. plantaginea* was sister to or distinctly separated from the remaining *Hosta* species, respectively (Figure 2 and Supplementary Figure 2). This separation agrees with its geographic origin (China) and its unique floral characteristics. *Hosta plantaginea* is the only species which blooms at night, has a fragrance, and possesses the largest flower among *Hosta* species with white perianths and yellow anthers. The genomic distinctness of *H. plantaginea* was shown earlier based on RAPD analysis (Sauve et al., 2005) and by means of having the largest genome size among this seven-species group (Zonneveld and Van Iren, 2001). Therefore, the placement of *H. plantaginea* in its own subgenus (i.e., *Hosta*) seems to be reasonable (Maekawa, 1940; Fujita, 1976).

The remaining species belong to subgenus *Bryocles*, among which either *H. yingeri* or *H. clausa* may be sister to all other taxa (Figure 2). Both ML trees retrieved six species in South Korea, but they were not all monophyletic due to the inclusion of *H. ventricosa*. Unlike other *Hosta* species, *H. ventricosa* is a tetraploid species, native to southern China, but it has been widely cultivated in China and western countries as a medicinal plant and as an ornamental, respectively (Mimaki et al., 1997; Yang et al., 2017; Chu et al., 2019). Previous studies suggested that *H. ventricosa* might have shared a common ancestor with *H. clausa* during their evolutionary history based on pollen morphology (Chung and Jones, 1989), DNA content (Zonneveld and Van Iren, 2001), and chromosome shape (Kaneko, 1968). This inference was supported in the nuclear ML tree, but not in the plastome ML tree which showed that *H. ventricosa* and *H. capitata* might have shared their maternal parent (Figure 2B). Since only two cultivated *H. ventricosa* accessions were included in this study, more samples of *H. ventricosa*, both natural and cultivated, should be included in future phylogenetic analysis to disentangle relationships among these three species.

In the nuclear ML tree, *H. yingeri* was sister to all other Korean taxa although its position was not fully supported. In contrast to the high BS and LPP support values, gCF and sCF values showed high incongruence among gene trees for its placing (Figure 2A). *Hosta yingeri* is endemic to Heuksan Island in South Korea, which is 97 km from the mainland, and is easily distinguishable from other *Hosta* species based on its floral traits, for example, a funnel-shaped flower with distinct stamens (3 + 3) and persistent bract at flowering (Jo and Kim, 2017). However, in the plastome ML tree, all five accessions of *H. yingeri* were sister to *H. jonesii*, and the genetic diversity between these two species was very low (0.026%; Table 1). This result indicates these two species might have shared their maternal parent, or that *H. yingeri* might have captured the plastome of *H. jonesii* through introgressive hybridization. Based on the divergence times estimated, *H. jonesii* and *H. yingeri* might share a most recent common ancestor (MRCA) 6.59 Ma, which corresponds to the late Miocene (Table 3). Also, considering their distribution (Figure 1), they might previously have had close contact. Therefore, the isolation of *H. yingeri* at Heuksan Island for a long period might have facilitated its morphological and nuclear differentiation. The hybridization experiments between *H. yingeri* and other Korean *Hosta* species resulted in less than 60% of pollen viability (Zonneveld and Van Iren, 2001), supporting its reproductive isolation along with its unique floral morphological features. Reproductive isolation is commonly found in island species (reviewed in Crawford and Archibald, 2017), which often exhibit incongruence between nuclear and plastome markers (French et al., 2016; Hughes et al., 2018; Waselkov et al., 2018; Thureborn et al., 2019). In addition, its genome size is much more similar to that of *H. capitata* and *H. clausa*, when compared to *H*. jonesii (Zonneveld and Van Iren, 2001), in agreement with their evolutionary relationships, as shown in the nuclear tree (Figure 4). Therefore, along with its unique floral characteristics, *H. yingeri*’s geographical isolation and its genome size support its position sister to all other Korean *Hosta*, rather than a sister relationship to *H. jonesii*.

The position of the *H. clausa* and *H. capitata* clades showed disparity between our two trees (Figure 2), as stated above. In the nuclear tree, *H. capitata* was sister to the remaining four species, while *H. clausa* was sister to these four species in the plastome tree (Figure 2). These two species are morphologically distinct. For instance, *H. capitata* and *H. clausa* can be distinguished because the former has a head-like inflorescence and short creeping rhizome, while the latter has a spike-like inflorescence and rhizome with underground stolon. Observed gene tree conflicts in both nuclear and plastome trees suggests either a lack of phylogenetic signal or incomplete lineage sorting (ILS) in Korean *Hosta* species may be at work. Their ambiguous phylogenetic relationships could thus be explained in terms of their rapid diversification during a relatively short period, as implied by the short branches in both trees and the overlapping divergence times estimated (Figures 2, 4).

The last three species, *H. jonesii*, *H. minor*, and *H. venusta*, formed a clade and their close relationship has been suggested in previous studies (Chung, 1991; Chung et al., 1991a,b). These three species have similar genome size, of about 17.5 pg (Zonneveld and Van Iren, 2001), and their interspecific nucleotide diversity was inferred to be very low (Table 1). Indeed, their low genetic diversity implies close relatedness and shared evolutionary histories, which are further supported by their morphological traits, such as ovate-to-lanceolate leaf blade, bract state at flowering, fruit color, and intermediate size of pistil and anthers (Jo and Kim, 2017). Especially, *H. venusta* accessions failed to form a clade, but were instead nested in the *H. minor* clade (Figure 2). The monophyletic group containing both *H. minor* and *H. venusta* was supported by only six gene trees out of 246 (Supplementary Figure 3), suggesting a high degree of underlying gene tree conflict or a lack of phylogenetic signal for the nuclear dataset. The latter could be the case, considering the genetic diversity between these two purported species (0.94% in the nuclear data, 0.004% in the plastome data) (Table 1). The genetic differentiation between these two purported species was also extremely low, 0.0868 and 0.2123 in nuclear and plastome datasets, respectively (Table 2). These values are much lower than those for other species, for example, the genetic differentiation between *H. jonesii* and *H. minor* was three to four times higher, compared to that between *H. venusta* and *H. minor* (0.2934 in nuclear data and 0.8372 in plastome data; Table 2). Thus, *H. minor* and *H. venusta* may be the same species, with a *F*_*st*_ < 0.25, and given the threshold previously estimated for species and populations from 97 studies of pairs of closely related species and populations (Hey and Pinho, 2012). Consequently, *H. venusta*, endemic to Jeju Island, should be recognized as a variety of *H. minor*, since they also present overlapping morphological characteristics (Jo and Kim, 2017), along with their low genetic diversity as shown here. However, there seems to be clinal variation in *H. venusta* along altitudes in Mt. Halla, Jeju Island (C. E. Lim, pers. comm.). Therefore, further analysis with more accessions of *H. minor* and *H. venusta* species from Jeju Island should be carried out to determine its taxonomic rank. Regardless of the species entity of *H. venusta*, the dispersal of *H. minor* from mainland Korea to Jeju Island might trace back to as early as 2 Ma when Jeju island was formed through volcanism (Woo et al., 2013).

*Hosta jonesii* occurs in Korea’s southern islands and its close relationship to *H. minor* was proposed in previous studies (Chung, 1991; Chung et al., 1991a,b). This study also supports their close phylogenetic relationship, but two accessions of *H. jonesii* were found to contain the plastome of *H. minor* (Hosjon_544677, Hosjon_15354) (Figure 2). Since all accession of *H. jonesii* belong to one clade in the nuclear ML tree, this cytonuclear discordance between the nuclear and plastome ML trees might be the result of introgressive hybridization between *H. minor* and *H. jonesii*. We observed these two accessions formed a clade with *H. minor*/*H. venusta* in some trees, indeed supporting a possible introgression (Supplementary Figure 4). These two species share their geographical distribution (Figure 1), thus, their close contact might have enabled their hybridization.

*Hosta* is well known for extensive hybridization due to its cultivar development, but no previous study has managed to detect hybridization events in nature. Earlier studies on the Korean *Hosta* species also failed to detect hybridization, since they examined either nuclear or plastid markers, focusing only on a small portion of the genome. Considering the low genetic diversities among *Hosta* species, the lack of information might be the main barrier to uncover hybridization events. In this study, we were able to reveal several hybridization events based on 246 nuclear coding genes and plastome sequences. Thus, this study suggests that a more genome-wide search is required for species groups with low genetic diversity to detect any hybridization or introgression events.

### Taxonomic Implications and Genome Size Evolution in Korean *Hosta* Species

A well-resolved phylogeny provides a backbone for investigating various biological aspects, such as taxonomic treatment and the evolution of morphological traits and genome sizes for a given lineage of interest. As for the taxonomic implications, this work showed that all six Korean species and *H. ventricosa* belong to subgenus *Bryocles*, since they form a monophyletic group. However, their placement into sections shows some disparities compared to previous treatments. *Hosta ventricosa* was assigned to section *Bryocles* due to its tetraploid status (Maekawa, 1937), while the rest of species are diploid. However, in our trees *H. ventricosa* forms a clade with *H. clausa*, which is a member of section *Stolonifera*, along with *H. yingeri* and *H. capitata.* Additionally, section *Stolonifera* was not supported by this study, as its three species were found to be paraphyletic (Figure 2). Based on the phylogeny obtained here, we can recognize three sections that consist of *H. yingeri* only, *H. capitata* only, and both *H. clausa* and *H. ventricosa*, respectively. The remaining three species of *H. minor/H. venusta* and *H. jonesii* were previously included in section *Lamellatae* (Zonneveld and Van Iren, 2001), in agreement with our reconstruction. However, the other two Japanese species, *H. tibae* and *H. tsushimensis*, should be examined to support the legitimacy of section *Lamellatae*. In addition, the legitimacy of subgenus *Bryocles* should also be evaluated by including Japanese *Hosta* species, most of which are thought to belong to subgenus *Giboshi* (Zonneveld and Van Iren, 2001).

Previously, it was proposed that the ancestral *Hosta* nuclear DNA content might be close to either 17 or 19 pg, and the speciation of *Hosta* might have been accompanied by increases in DNA content in dry habitats and by decreases in wet environments (Zonneveld and Van Iren, 2001). However, this idea contradicts recent works showing increased genome sizes could be associated with early flowering and a tendency to grow in wet environments (Knight et al., 2005; Vesely et al., 2012). Also, under nutrient-limited conditions species with large genomes would be less competitive than species with small genomes, as they would need more resources to maintain their large genomes (Smarda et al., 2013; Guignard et al., 2016). The ancestral state estimation for the genome size, given our *Hosta* species tree, shows that the MRCA of *H. plantaginea* and the remaining species might have had a large genome size (19.9 ± 1.1 pg), supporting Korean *Hosta* species may have diversified by reducing their DNA content (Figure 3). For example, *H. yingeri*, which is sister to all the remaining Korean *Hosta* species and *H. ventricosa*, has 19 pg of 2C value, while *H. minor*, which is furthest from the root, contains 17 pg of 2C value (Zonneveld and Van Iren, 2001). Although there is a trend of DNA content decrease in Korean *Hosta* species, there were substantial overlaps in confidence intervals among species. This might be explained by their rapid diversification in similar environmental conditions in South Korea, but the relationship between genome size and their ecological and evolutionary constraints should be further tested. Therefore, the inclusion of Japanese *Hosta* species that occur in diverse conditions could shed light on the speculation of the effect of genome size on *Hosta* speciation. The better resolved tree with Japanese taxa should provide a backbone for more accurate inference on the genome size evolution in *Hosta*.

### Divergence Time Estimates for the *Hosta* Crown Group and Korean *Hosta* Species

Previous analysis of 69 plastome protein-coding genes estimated that *Hosta* diverged at the crown node of the ABK clade during the Oligocene (20–27 Ma) (McKain et al., 2012, 2016). However, our analysis with more *Hosta* species estimated its divergence during the late Eocene (38.38 Ma). Since our estimates for other lineages are not much different from the previous ones (Table 3), analysis with more *Hosta* taxa and with more genes (i.e., 79 plastome protein-coding genes) might result in different estimates for *Hosta*. Here, we estimated divergence times for the *Hosta* crown group and for Korean *Hosta* species for the first time. Particularly, 95% HPD intervals were very wide for *Hosta* clades (Figure 4 and Table 3), which might be due to the low sequence divergence among *Hosta* species, as shown by their short branch lengths (Supplementary Figures 1, 2) and low nucleotide diversities (Table 1). Although our estimates were not narrowed down to specific time points, the estimated times for *Hosta* species suggest rapid diversification of Korean *Hosta* species over a short period (∼ 10 Ma) during the Miocene.

During the early Miocene, the Japanese Islands started their separation from the continent, and this process was completed during the middle Miocene and resulted in the formation of the East Sea (Yabe et al., 2019). The divergence time we estimated for crown Korean *Hosta* dates back to the late Miocene, when the Korean Peninsula and the Japanese Islands were completely separated by the East Sea. Thus, we hypothesize that limited gene flow between Korean and Japanese *Hosta* species might have accelerated the speciation of *Hosta* species in South Korea, without necessarily increasing their genetic diversity. However, the phylogeny of *Hosta* without Japanese species does not allow to assess whether Korean *Hosta* evolved from Chinese vs. Japanese taxa. The latter case would involve the migration of *Hosta* species from China to either southeastern Russia-to-Japan or Japan and then onto South Korea. Therefore, it is critical to include Japanese *Hosta* species, which have the highest species diversity of *Hosta* (ca. 20 species), as well as Russian accessions, to figure out the origin of the Korean species.

### Application of Hyb-Seq Results for Identifying the Origin of Cultivar Species

*Hosta* species have very showy foliage and shade tolerance, so they have considerable economic value as landscaping and ornamental plants. Their popularity, in turn, has brought about a long history of cultivation and extensive hybridization, resulting in many extant cultivars. However, their origins were not clearly recorded in many cases. Here, we tried to evaluate whether 246 single-copy nuclear genes and complete plastome sequences were useful for inferring the origin of cultivars. In this study, we examined nine cultivars, two *H. capitata*, three *H. venusta*, and four *H. clausa* cultivars, for which their origins are known. All nine cultivars were clustered with their corresponding parent species, but our results suggest that, for a given species, cultivars could have been developed from different individuals from different geographic regions. For example, two *H. clausa* cultivars (Hoscla_15 and Hoscla_16) were close to Hoscla_15327 collected from southern South Korea, while the other two cultivars were clustered with *H. clausa* species occurring in northern South Korea (Figure 1). Also, the results for the *H. venusta* cultivars show the importance of a well-resolved phylogeny in inferring the origin of cultivars. Although two *H. venusta* cultivars (Hosven_32, Hosven_33) formed a clade sister to *H. venusta* (Hosven_15311), Hosven_34 was sister to *H. minor* (Figure 2), not *H. venusta*. Considering these two species may instead be one and the same (see above), this placement is not surprising. Therefore, a well-resolved and supported phylogeny is critical to infer the origin of cultivars as well as the provision of genomic resources. Although our work shows the usefulness of these 246 single-copy nuclear genes and complete plastome sequences for exploring the origins of cultivars, these genomic resources should be further tested with more cultivars of known and unknown origins. Then, an extensive investigation can lead to the development of molecular markers useful for inferring their origin, which could be employed for evaluating whether *Hosta* species currently growing in a garden originated from South Korea, Japan, China, or even Russia. Also, those molecular markers could be used for cataloging cultivars, which is critical for the conservation of natural species. Finally, *Hosta* breeding programs will benefit from their known parentage in developing new cultivars.

## Conclusion

The Hyb-Seq method allowed us to capture several hundreds of low-to-single-copy nuclear genes as well as to obtain full plastomes. Due to low genetic diversities, commonly used nuclear and plastome markers have shown inconsistent results with low resolution in the phylogeny of *Hosta*. Here, the genomic data obtained with Hyb-Seq successfully helped identify Korean *Hosta* species and permitted inferring a well-resolved and supported phylogeny. In addition, we detected several putative introgressive hybridization events, never before found. However, ILS could not be ruled out due to the high incongruence observed among nuclear genes. This gene tree discordance might result from a lack of phylogenetic signal for Korean *Hosta* species following their rapid diversification during a short time interval. Last, we demonstrated the applicability of genomic data from Hyb-Seq for tracing the origin of cultivated *Hosta* species. In summary, this study provides the most comprehensive genomic resources to date for phylogenetic inference, the development of potential molecular markers for population and conservation studies of *Hosta*, as well as for unraveling the origin of many cultivars.

## Data Availability Statement

The datasets presented in this study can be found in online repositories. The names of the repository/repositories and accession number(s) can be found in the article/Supplementary Material.

## Author Contributions

CL designed the project. CL and B-YL conceived ideas and prepared funding and samples. SK oversaw the procedure of data generation. M-JY analyzed the data and wrote the manuscript. All authors have read and approved the manuscript.

## Conflict of Interest

The authors declare that the research was conducted in the absence of any commercial or financial relationships that could be construed as a potential conflict of interest.

## Footnotes

1 http://www.geneious.com

2 https://github.com/mossmatters/MJPythonNotebooks

3 https://doi.org/10.6084/m9.figshare.14043965

4 http://tree.bio.ed.ac.uk/software/figtree/
