# Supplementary material for: Phylogenomics With Hyb-Seq Unravels Korean Hosta Evolution
Source: Front Plant Sci. 2021 Jul 8;12:645735. doi: 10.3389/fpls.2021.645735 (PMC8296909; doi:10.3389/fpls.2021.645735)
Supplement: Supplementary file 1 [file Presentation_1.PPTX]

## Slide 1
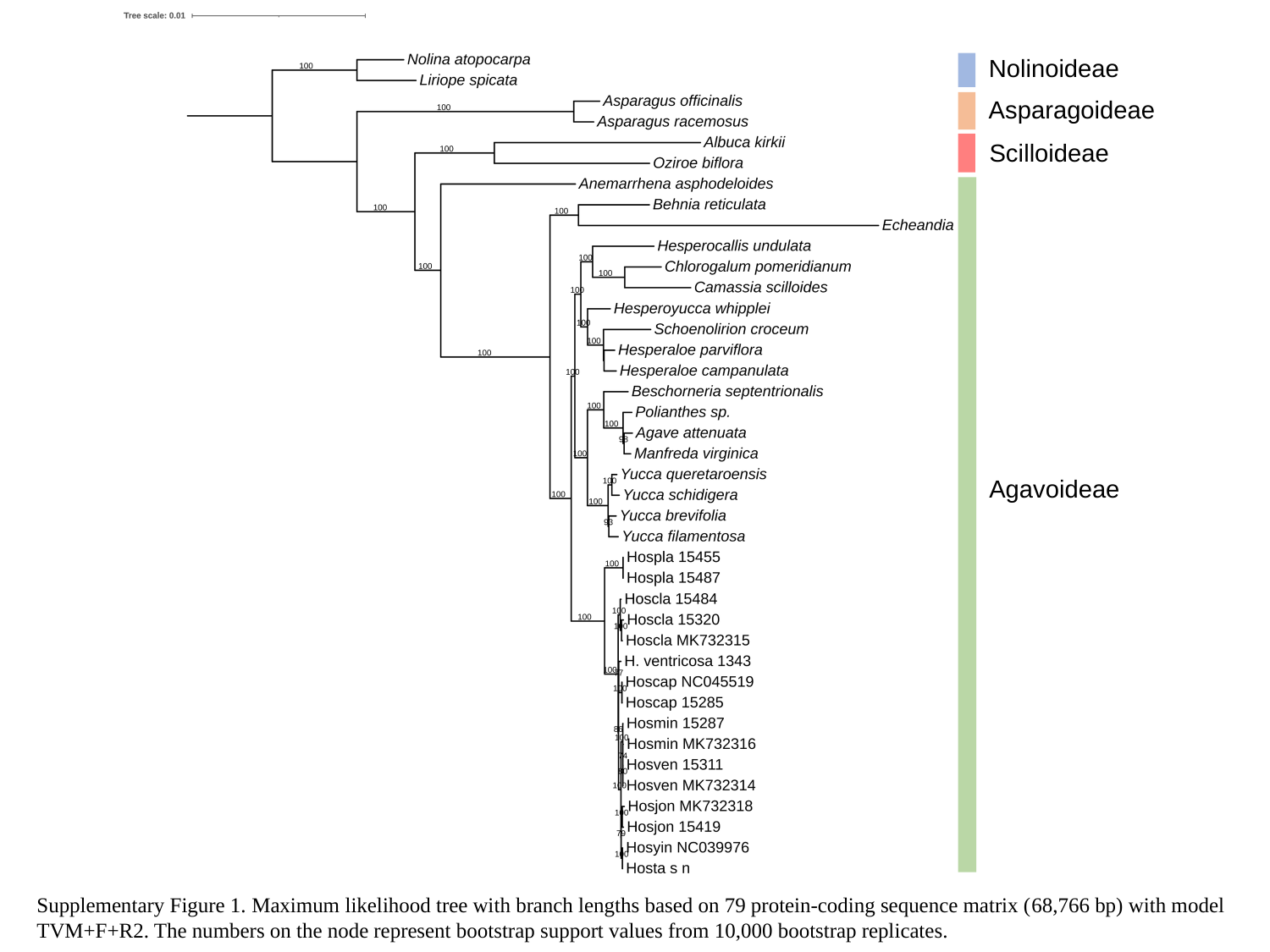

Nolinoideae
Asparagoideae
Scilloideae
Agavoideae
Supplementary Figure 1. Maximum likelihood tree with branch lengths based on 79 protein-coding sequence matrix (68,766 bp) with model TVM+F+R2. The numbers on the node represent bootstrap support values from 10,000 bootstrap replicates.
