# Supplementary material for: Phylogenomics With Hyb-Seq Unravels Korean Hosta Evolution
Source: Front Plant Sci. 2021 Jul 8;12:645735. doi: 10.3389/fpls.2021.645735 (PMC8296909; doi:10.3389/fpls.2021.645735)
Supplement: Supplementary file 2 [file Presentation_2.PPTX]

## Slide 1
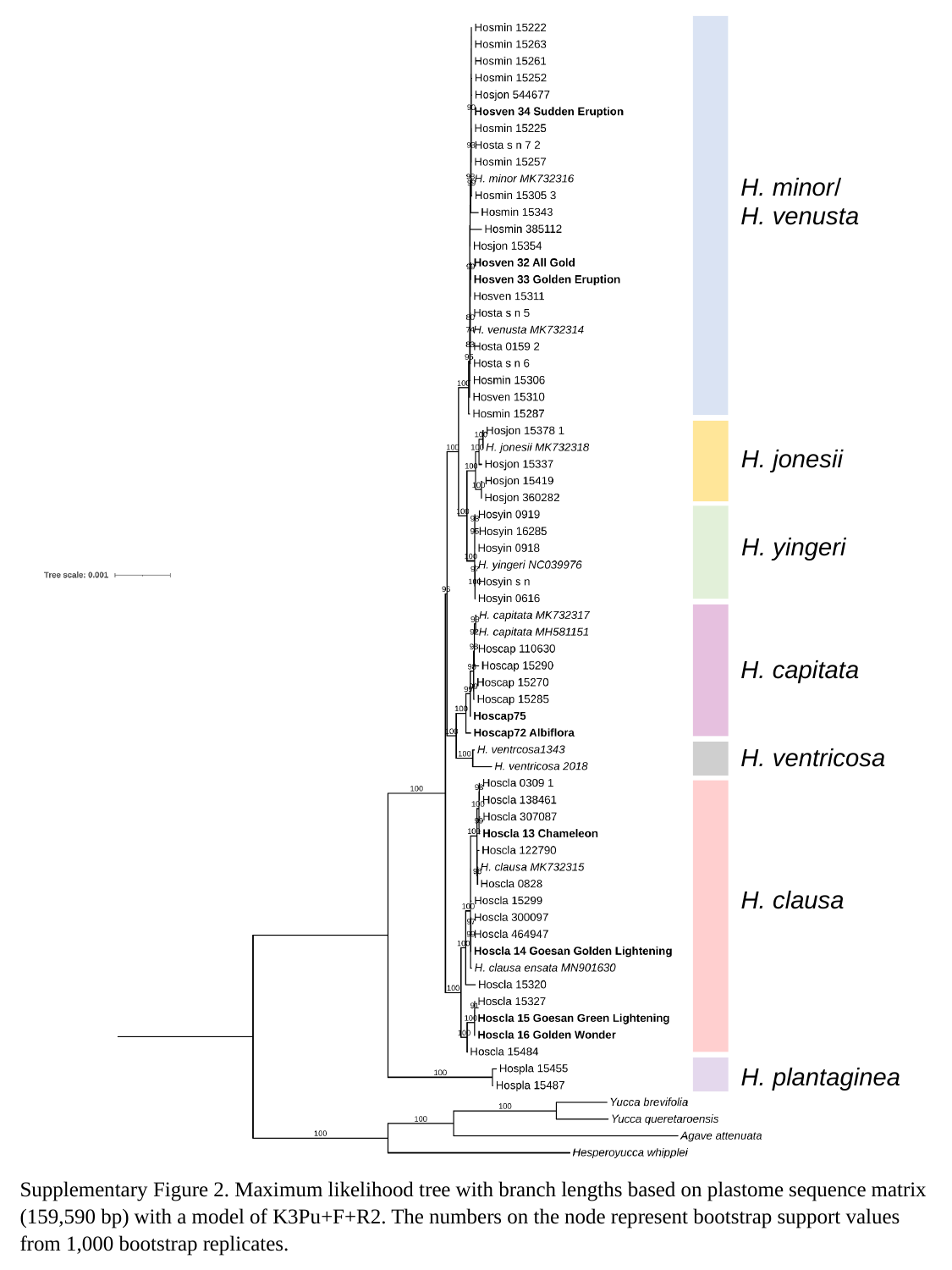

H. minor/
H. venusta
H. jonesii
H. yingeri
H. capitata
H. ventricosa
H. clausa
H. plantaginea
Supplementary Figure 2. Maximum likelihood tree with branch lengths based on plastome sequence matrix (159,590 bp) with a model of K3Pu+F+R2. The numbers on the node represent bootstrap support values from 1,000 bootstrap replicates.
