# Supplementary material for: Phylogenomics With Hyb-Seq Unravels Korean Hosta Evolution
Source: Front Plant Sci. 2021 Jul 8;12:645735. doi: 10.3389/fpls.2021.645735 (PMC8296909; doi:10.3389/fpls.2021.645735)
Supplement: Supplementary file 3 [file Presentation_3.PPTX]

## Slide 1
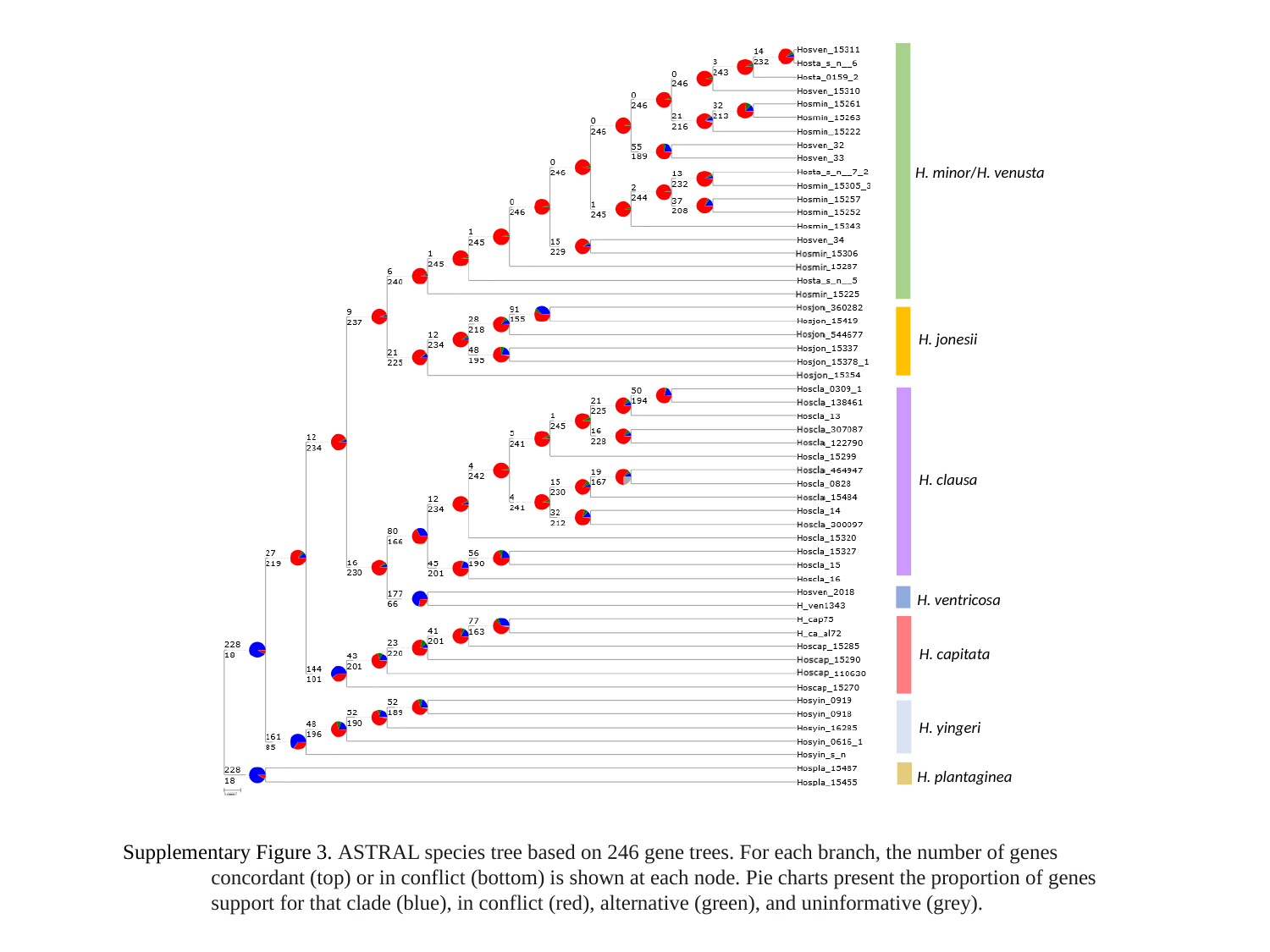

H. minor/H. venusta
H. jonesii
H. clausa
H. ventricosa
H. capitata
H. yingeri
H. plantaginea
Supplementary Figure 3. ASTRAL species tree based on 246 gene trees. For each branch, the number of genes concordant (top) or in conflict (bottom) is shown at each node. Pie charts present the proportion of genes support for that clade (blue), in conflict (red), alternative (green), and uninformative (grey).
