# Supplementary material for: Phylogenomics With Hyb-Seq Unravels Korean Hosta Evolution
Source: Front Plant Sci. 2021 Jul 8;12:645735. doi: 10.3389/fpls.2021.645735 (PMC8296909; doi:10.3389/fpls.2021.645735)
Supplement: Supplementary file 4 [file Presentation_4.PPTX]

## Slide 1
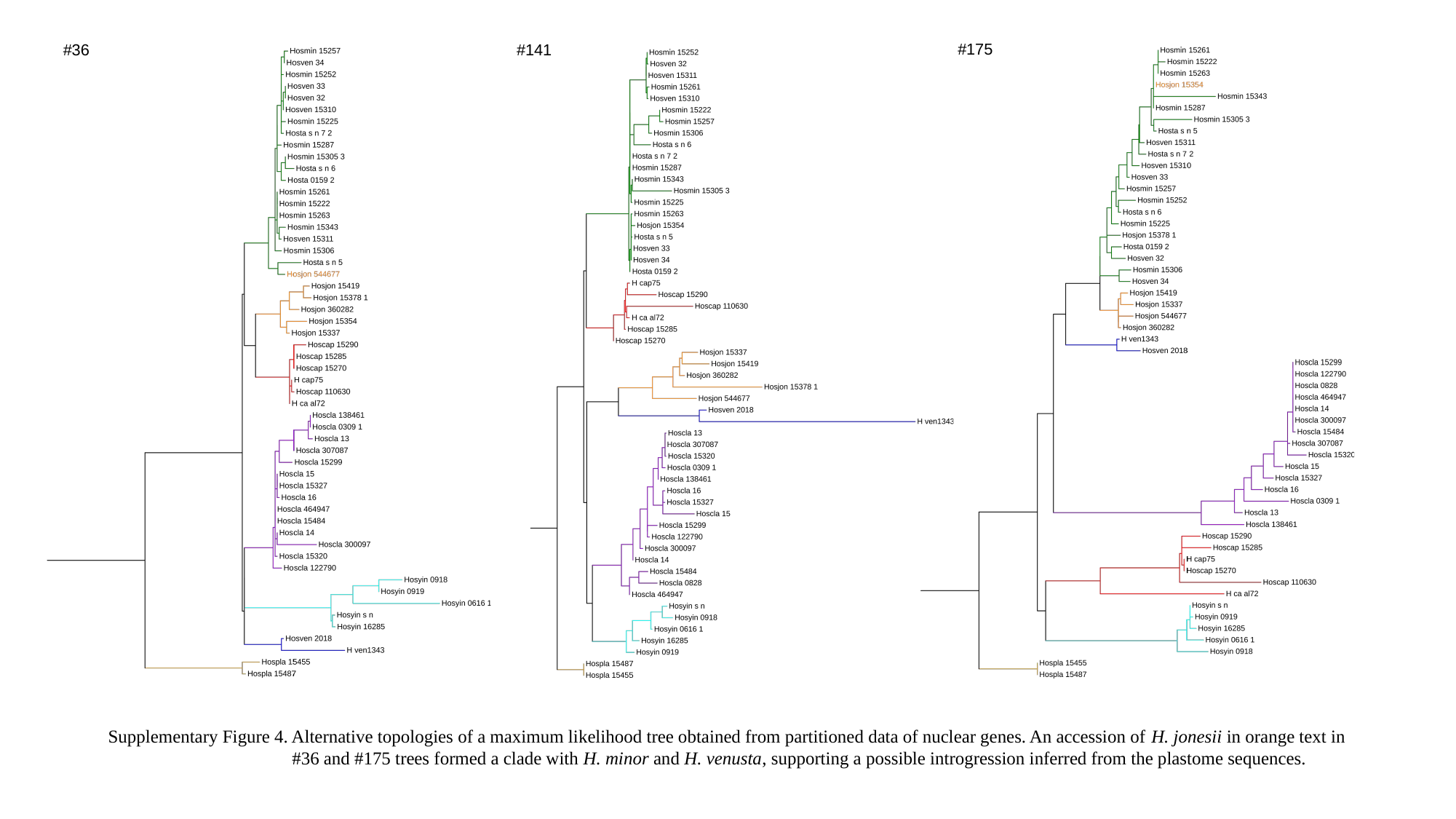

#175
#36
#141
Supplementary Figure 4. Alternative topologies of a maximum likelihood tree obtained from partitioned data of nuclear genes. An accession of H. jonesii in orange text in #36 and #175 trees formed a clade with H. minor and H. venusta, supporting a possible introgression inferred from the plastome sequences.
